# Developing Professional Identity in Undergraduate Pharmacy Students: A Role for Self-Determination Theory

**DOI:** 10.3390/pharmacy5020016

**Published:** 2017-03-24

**Authors:** Martina F. Mylrea, Tarun Sen Gupta, Beverley D. Glass

**Affiliations:** James Cook University College of Medicine and Dentistry, Townsville 4811, Queensland, Australia; tarun.sengupta@jcu.edu.au (T.S.G.); beverley.glass@jcu.edu.au (B.D.G.)

**Keywords:** professional identity, pharmacy education, health professionals, professionalisation, self-determination theory

## Abstract

Professional identity development, seen as essential in the transition from student to professional, needs to be owned by the universities in order to ensure a workforce appropriately prepared to provide global health care in the future. The development of professional identity involves a focus on who the student is becoming, as well as what they know or can do, and requires authentic learning experiences such as practice exposure and interaction with pharmacist role models. This article examines conceptual frameworks aligned with professional identity development and will explore the role for self-determination theory (SDT) in pharmacy professional education. SDT explains the concepts of competence, relatedness and autonomy and the part they play in producing highly motivated individuals, leading to the development of one’s sense of self. Providing support for students in these three critical areas may, in accordance with the tenets of SDT, have the potential to increase motivation levels and their sense of professional identity.

## 1. Introduction

A 2010 report by an independent commission on the education of health professionals for the 21st century called for a renewed approach to health education, one which promoted professionalism and in particular the development of a professional identity [1]. The report came amidst an era where professions and society in general have seen a general decline in professionalism [2,3,4]. In Australia, academics in health observe mandatory reporting on student conduct and performance to the Australian Health Practitioner Regulation Agency [5]. As a result, there is increased expectation that university health degrees provide opportunities for student professional development [6].

A shift is occurring with the suggestion that becoming a professional requires more than the acquisition of appropriate attitudes, values and behaviors, that it is a complex process involving the formation of a professional identity. Health education research suggests that the development of a professional identity is essential during the transition from student to professional [7,8,9,10]. Cruess et al. emphasize that students need the opportunity to “think, act and feel” like a professional during their studies [7].

In pharmacy education, however, research has indicated that identity development has been neglected in pharmacy curricula [8], often being deferred until the final stages of the degree [11]. Several authors have defined professional identity [12,13] with Skorikov and Vondracek highlighting the interplay between human motivation and relevant skills and knowledge. They state that a professional identity is represented by:

‘a complex structure of meanings in which the individual links his or her motivation and competencies with acceptable career roles’.

[14]

A recent review of identity development in higher education by Trede reflects the position of the independent commission on the education of health professionals [1], where Trede states that ‘Universities need to claim their role in professional identity development to prepare graduates for global citizenship, leadership and future practice’ (p. 379) [15].

This paper presents a discussion around professional identity development in health education broadly and pharmacy education in particular. Conceptual frameworks currently aligned with professional identity development will be explored. Finally we propose self-determination theory [16,17], a motivation-based theory, as a theoretical framework for professional identity development in pharmacy tertiary education.

## 2. Professional Education and Identity

Researchers in medical education have been particularly active in the area of identity development with Cruess et al. recently stating that professional identity should be the foundation of professionalism [18]. Dawodu and Rutter have highlighted that the responsibility of professionally preparing students has increasingly fallen onto academic educators [19]. There is, however, a lack of research into professional identity development in tertiary education [15] and as a result there is little understanding of how to develop such an identity within each student. Research into the development of effective teaching approaches that foster professional identity formation is therefore needed [7,15].

Trede et al. state that for identity development to occur, learning must happen through direct contact with the practice [15]. Experiential education with its exposure to practicing professionals and patients is essential for student identity development [20]. While work placements are regarded as the ideal approach for student immersion in the profession [6], in reality schools of pharmacy face shortages of clinical placements [21], which may also deliver inconsistent experiences amongst students [6,22]. In an effort to increase exposure to practice, some courses have turned to technology, bringing virtual clinical placements into the curriculum [22]. Practical classes such as compounding and clinical dispensing sessions as well as exposure to professional role models such as patient- facing pharmacists/academics, have also been identified as particularly effective strategies for student professional development [23]. According to Reid et al., it is the authenticity of the learning experiences which is important for successful identity development [24].

Dall’Alba describes becoming a professional as a transformation that is unique to each individual [25]. She warns that educational programs failing to acknowledge and provide support for this transition may contribute to a ‘crisis of confidence in professional education’ (p. 136) [25]. This crisis relates to the inadequacy of professional education programs to train students to cope within the modern healthcare context. She cites advances in information technology, new conceptualisations of interdisciplinary contexts and changing knowledge as contributing to the rapidly evolving professional landscape. Dall’Alba states that a focus on skills and knowledge alone is insufficient to adequately prepare the student to cope with this challenging and dynamic setting. She also explains that the current theory–practice gap, which exists in many programs, prevents students from thinking as, acting as or being a professional [25]. With a similar view, Cruess et al. explain that while professional attitudes, values and behaviours are essential to the developing professional, they do not account for the entire process behind becoming professional [7]. They believe that this occurs through a process of internalisation, where the student comes to ‘think, act and feel’ like a professional [7]. In order to achieve this they state that the development of a professional identity should be one of the main goals of a medical educational program [7]. The concept of professional identity development is emerging as a complementary and perhaps crucial element to meaningful and lifelong professional growth [7,26,27].

It has been established that curriculum design can exert a significant influence on student professional identity development [6,25,27] so it is important to consider educational strategies for identity development [7]. As Reid et al. explain, student learning trajectories ‘are influenced by the manner in which the sense of the profession is communicated and articulated through the design and pedagogy of the educational program’ (p. 734) [27]. Adams et al. suggest that the task for educators is to find a framework for educational programs so that they can best prepare students for their professional role [28].

Optimal conditions for identity development should use an integrated approach whereby experiences on placement are addressed and reinforced as part of curriculum coursework [6,29]. Branch stated that a combination of teaching methods should be employed over an extended period across the curriculum. Approaches including experiential learning, critical reflection and small-group teaching should feature in curriculum design as a combined model and importantly extended long term throughout the course structure [30]. While these strategies are well-supported in the literature, a sound theory-based approach to identity development is needed, one which will translate through to effective teaching strategies at the classroom level. Research clearly supports introducing concepts surrounding professionalism and professional identity early in the course structure, thus providing students ongoing opportunities to relate to and engage with the profession [7,9,23,31].

## 3. Pharmacy Education and Identity

Professional identity development is a concern at the highest level of pharmacy education policy development. The International Forum for Quality Assurance of Pharmacy Education was established by the International Pharmaceutical Federation (FIP) in 2001. In their 2014 framework for the quality assurance of pharmacy education [32], they quote the World Health Organisation’s World Health Report [33], which espouses the benefits of authentic, practical experiences for health students. The report states that students who are exposed to the practice experience:

‘an increase in empathy towards people with illnesses, have greater self-confidence and professional identity, and have learned effectively from the knowledge, attitudes, values, behaviours, and judgments of experienced practitioners’.

(p. 48) [33]

A number of researchers have reported on the essential role of experiential learning in developing pharmacy students as professionals [6,11,34]. Stupans and Owen report particularly on the importance of planned integration of practice-based learning within the overall curriculum [6] The Schafheutle review of pharmacy schools in the UK identified role models and exposure to the practice as critical elements in the professional development process [31]. Contact with practicing pharmacists in the placement context was identified as most influential [23]. A study by Harding and Taylor viewed both pharmacist academics and practicing pharmacists as important role models for the professional socialisation of students and reported on the high regard students have for the presence of practicing pharmacists during their studies [35]. A threat to this enrichment is the declining numbers of full-time practicing pharmacists in pharmacy schools, which limits the access students have to practicing professionals [11]. To address this many schools have resorted to part-time con-joint appointments to maintain the presence of practicing pharmacists.

Research carried out at an Australian university examined the role of curriculum in professional identity development. Noble et al. utilized a qualitative ethnographic study to explore the views of students across all years of an undergraduate pharmacy course [8]. The research revealed that there were few opportunities where students could explore their professional identity. There were limited opportunities for interactions with pharmacist role models and little reflection on the progress towards their identity development. The authors saw these as lost opportunities in the curriculum. They commented on the importance of starting a dialogue around what it means to be a pharmacist, and to provide opportunities for interactions between students, pharmacists and patients. Providing feedback on student submitted work was also regarded as an effective strategy for fostering identity development [8], a result supported by other studies [36,37]. Feedback in the form of evaluation against internal standards, or provided by practicing pharmacists and educators, is important for student experience and reflection. Feedback provides the validation or confirmation needed by the student throughout the identity formation process [8].

The importance of professional identity development in pharmacy education is beginning to emerge [8,31,35,38]; however, it is commonly deferred until the final stages of the course once required scientific foundations such as chemistry and physiology have been covered [35]. This removes the practice of pharmacy from being patient-centered in the early stages of the course, moving to a sole focus on, for example, the chemistry and biological foundations of pharmacy [11]. A consequence of this is delayed professional acculturation and the diminution of the importance of the professional role. This is in contrast to more recent thinking by those who believe that professional education must begin at the outset of the course and continue for the duration of study [30,39,40].

## 4. Theoretical Frameworks

In their review of professional identity development in higher education, Trede et al. commented on the wide variety of theoretical frameworks applied to this area. There is unfortunately a lack of consensus amongst researchers as to the most effective approach to professional identity development [15]. Their review reported theories including Wenger’s theory of communities of practice and situated learning [41], a theory often quoted in the area of professional education. Learning theories such as Schon’s reflective practice [42] and Mezirow’s critical reflection [43] have also appeared in studies relating to professional identity.

Early work by Merton [44] referred to professional identity as developing through thinking, acting, and feeling like a member of the profession. This work prompted Cruess et al. to define identity development as a stage dependent development of self [7]. Dall’Alba similarly supported the development of a ‘sense of being’, recommending the integration of epistemology and ontology as a framework for professional education [25]. She has stated that students will take a variety of trajectories on their professional development path, characterized by individual transition. 

To gain a better understanding of how students develop a sense of professional identity, Reid et al. [27] conducted a collaborative study between research groups in Sweden and Australia. They investigated student perceptions of how education and experiences of professional work contributed to their professional identity. From this study a model for professional identity development was formed. Reid et al. [24] proposed that professional identity development can be described as a function of two dimensions: ‘knowledge for the profession’ and ‘learning for professional work’. Successful intersection of these two dimensions results in connection with the profession and subsequent identity formation. The authors refer to the development of ‘sensitising dispositions’, an artefact of this process, which orients the student within their profession by placing them in context both professionally and personally. These dispositions represent the linking fabric between the two dimensions of their model and extend student professional learning beyond skills and knowledge to ontological aspects of professional practice [24], such as those proposed by Dall’Alba [25]. The authors made four recommendations for professional socialisation, calling them strategies for ‘blurring the boundaries’: (1) involve practicing professionals in higher education, (2) include students in work situations, (3) align learning with authentic work practices and (4) create opportunities for inter-professional learning.

The ultimate aim as described by the authors is the realisation and internalisation of a connection between their individual and professional self, otherwise defined as the ontological dimension of professional development [24]. This is clearly in agreement with the work of Dall’Alba in her description of individual trajectories towards becoming a professional in a particular field. She states that educational programs often fail to accommodate these differences in their teaching approaches [25]. Holden et al. established a steering committee to develop a framework around which professional identity could be fostered and assessed. The framework identified six domains and thirty subdomains to capture the complexity of professional identity across three developmental phases: Transition, Early Developing Professional Identity and Developed Professional Identity [45]. Unlike the models previously discussed, Holden et al. offer practical teaching strategies and learning environments, which have been shown to promote professional identity development. Their model encourages the integration of their framework into existing curricular structures, thus capitalizing on established teaching and assessment approaches.

## 5. Self-Determination Theory

**Figure 1:**
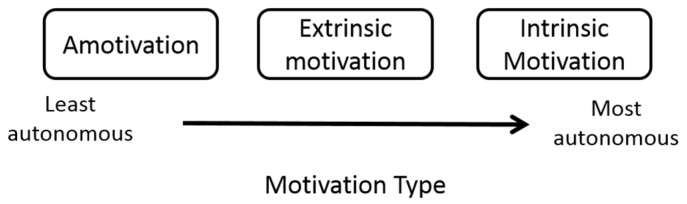
Diagrammatic representation of motivation types as described by Self-Determination Theory. Adapted from Ryan and Deci [47].

The absence of a theoretical basis for professional identity development in health programs is clear. An understanding of the mechanism behind human identity development is required, in order to accurately inform curriculum initiatives for the formation of professional identity. In the 1980s, psychologists Deci and Ryan from the University of Rochester, USA, developed a theory to explain identity development [46]. Self-determination theory (SDT), now long standing and widely applied, defined the role of motivation regulators in the formation and maintenance of identity. The authors describe human motivation as lying on a continuum that features three categories of motivation; amotivation, extrinsic motivation and intrinsic motivation, the latter representing the most autonomous state (Figure 1).

According to SDT, motivation is described as being absent (amotivation), driven by external forces such as monetary awards (extrinsic), or activated by internal forces (intrinsic), such that individuals willingly engage in the pursuit for interest or enjoyment. It is during this transition from external to internal regulation, where identity is internalized and becomes part of the individual’s sense of self [48]. Their theory proposes that high levels of motivation are supported through the satisfaction of three fundamental human psychological needs, or nutriments, namely competence, relatedness and autonomy [49]. Competence is the ability to demonstrate mastery in a particular area or in other words to have some effect on their surroundings [50]. Relatedness refers to the need to have connections with and to care for others [50] and autonomy refers to the propensity of an individual to self-organize experiences and actions, the authors making comparison to the concept of volition [50]. Individuals who experience support and growth in each of these three areas are more likely to have high levels of motivation and to develop and maintain the particular identity in question. High levels of motivation are also associated with success and wellbeing [51,52]. The more an identity is internalized ‘the more it will represent a deeply held and flexibly enacted aspect of one’s identity and self’ (p. 262) [49].

## 6. Pharmacy Education and SDT

The use of SDT as a theoretical basis upon which to develop professional curricular initiatives in pharmacy education may be appropriate on three fronts. Firstly, the application of this theory may be well placed given the obvious role played by competence, relatedness and autonomy in professional pharmacy practice and in general healthcare. An examination of the conceptualization of professionalism by the American Association of Colleges of Pharmacy (AACP, will find descriptions of safe, efficacious and *competent* practice of the qualified professional, *relatedness* reflected in the fiduciary relationship between the pharmacist and the patient and *autonomous* behaviors such as decision making and self-education [53]. A similar comparison can be made with the Australian Code of Conduct [54] for registered health practitioners and the Code of Ethics for Pharmacists [55].

Secondly, SDT has already been identified as having particular relevance in the field of medical education [45,56,57,58] and thus could equally be seen to have application in pharmacy education. Ten Cate et al. have explored the opportunities for integrating SDT into medical school curricula. The value of SDT for medical students is seen as an opportunity to create motivation for learning by increasing their sense of competence, autonomy and relatedness. The authors highlighted curriculum structure, classroom teaching, assessments and clinical training as areas where the role of SDT could be expanded [56]. Professional identity development however was not addressed specifically, other than to say that it is affected by the learning environment. To increase the impact of SDT in the curriculum, Ten Cate et al. suggested stimulating motivation through student-centered education such as problem based learning and the use of small group teaching [56]. Both approaches enhanced student relatedness and autonomy by encouraging and acknowledging student opinion and input. Also of interest is the suggestion that professional identity development may be enhanced by giving students opportunities to be more autonomous and less controlled by academics in their approach to learning [56]. This might involve allowing students to make choices about timing of assessments and being coached in learning styles and self-regulation techniques. Inviting students to become assistant instructors was also seen to enhance feelings of competence and autonomy. Early patient contact and participation in professional workplaces, such as during experiential placements, particularly enhanced student competence, relatedness and autonomy [56]. Also working in medical education, Kusurkar et al. espouse the value of autonomy-supportive classroom teaching techniques for developing intrinsic motivation in medical students. The authors make 12 recommendations which include identifying student needs, encouraging active participation, constructive feedback, emotional support and allowing students to make choices about their learning [59].

**Figure 2:**
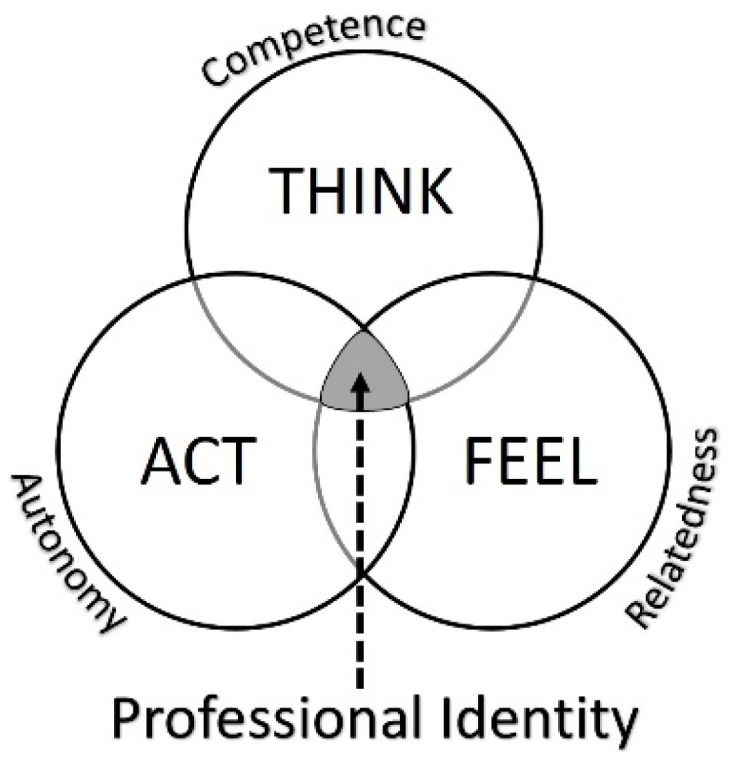
Conceptualisation of the role of self-determination theory nutriments in the development of professional identity.

Finally, approaching professional identity education from a proven theoretical framework such as SDT provides a foundation for curriculum design, which is based on an established mechanism for human identity development. Increased understanding of the motivational processes behind identity formation and integrated curricular support for the SDT nutriments (competence, relatedness and autonomy) may facilitate professional identity formation by assisting the student to “think, feel and act” like a pharmacist (Figure 2).

According to SDT, identity development is a result of a process of internalization, occurring as the student transitions from extrinsic through to an intrinsic state of motivation [17,47]. Recent publications exploring professional identity development refer to the importance of ‘internalization’ during the process of becoming a professional [7,24]. There appears to be an interesting synchronicity between SDT and professional identity development, one which may serve to enhance approaches to professional education. Therefore it is reasonable to postulate that by embedding the principles of SDT within pharmacy professional education, a positive influence on professional identity formation may be realized.

## 7. Conclusions

Adequately preparing students to cope with the demands of a changing and challenging health care setting requires the facilitation of professional identity development. Experiential learning through work placements, exposure to and interaction with practicing pharmacists and teaching sessions related to the practice are most effective for promoting identity formation. This paper proposes, in addition, a role for SDT as a theoretical framework for professional identity development in pharmacy education. An opportunity exists to apply this established theory in motivational psychology with its inherent link to identity development. Providing support for student competence, relatedness and autonomy within a professional pharmacy degree program may better prepare graduates for entry into the contemporary healthcare workplace.
